# Tea, Coffee, and Milk Consumption and Colorectal Cancer Risk

**DOI:** 10.2188/jea.JE20130063

**Published:** 2014-03-05

**Authors:** Chadwick John Green, Palina de Dauwe, Terry Boyle, Seyed Mehdi Tabatabaei, Lin Fritschi, Jane Shirley Heyworth

**Affiliations:** 1 School of Population Health, The University of Western Australia, 35 Stirling Hwy, Crawley WA 6009, Australia; 2 Western Australian Institute for Medical Research, The University of Western Australia, B Block, Hospital Avenue, Sir Charles Gairdner Hospital, Nedlands WA 6009, Australia

**Keywords:** epidemiological, tea, coffee, milk, colorectal cancer, risk factors

## Abstract

**Background:**

Data regarding the effects of tea, coffee, and milk on the risk of colorectal cancer are inconsistent. We investigated associations of tea, coffee, and milk consumption with colorectal cancer risk and attempted to determine if these exposures were differentially associated with the risks of proximal colon, distal colon, and rectal cancers.

**Methods:**

Data from 854 incident cases and 948 controls were analyzed in a case-control study of colorectal cancer in Western Australia during 2005–07. Multivariable logistic regression was used to analyze the associations of black tea (with and without milk), green tea, herbal tea, hot coffee, iced coffee, and milk with colorectal cancer.

**Results:**

Consumption of 1 or more cups of herbal tea per week was associated with a significantly decreased risk of distal colon cancer (adjusted odds ratio, 0.37; 95% CI, 0.16–0.82; *P_Trend_* = 0.044), and consumption of 1 or more cups of iced coffee per week was associated with increased risk of rectal cancer (adjusted odds ratio, 1.52; 95% CI, 0.91–2.54; *P_Trend_* = 0.004). Neither herbal tea nor iced coffee was associated with the risk of proximal colon cancer. Hot coffee was associated with a possible increased risk of distal colon cancer. Black tea (with or without milk), green tea, decaffeinated coffee, and milk were not significantly associated with colorectal cancer risk.

**Conclusions:**

Consumption of herbal tea was associated with reduced risk of distal colon cancer, and consumption of iced coffee was associated with increased rectal cancer risk.

## INTRODUCTION

Poor diet is a known modifiable risk factor for colorectal cancer (CRC),^1^^–^^4^ and many foods and beverages have been associated with increased or decreased risk of CRC.^5^ These beverages include tea, coffee, and milk, which are among the most commonly consumed beverages in the world.

Tea leaves are a source of flavonoid antioxidants—a polyphenol subgroup.^6^^,^^7^ The polyphenol concentration is highest in green tea.^8^^,^^9^ Previous reviews have shown that polyphenols inhibit tumor invasion, cell proliferation, and cell transformation, and induce tumor apoptosis.^7^^,^^10^ However, observations from epidemiologic studies are inconsistent regarding the role of black and green teas as risk factors for CRC.^11^^–^^13^ Herbal tea, ie, any infusion made solely from herbs, flowers, or other material of organic origin that does not contain leaves from the tea bush, is also high in polyphenol antioxidants.^14^ Although 2 case-control studies found an inverse association between herbal tea and breast cancer risk,^15^^,^^16^ to our knowledge the effect of herbal tea on CRC risk has not been investigated.

Coffee has an antioxidant capacity up to 8 times that of an equal volume of tea, depending on the preparation method used.^17^ Unfiltered coffee also contains 2 important diterpenes—cafestol and kahweol—which may reduce cancer risk by altering the way the body metabolizes carcinogens.^18^^–^^20^ Conversely, coffee may function as a carcinogen by raising plasma homocysteine levels^21^ or by increasing hydrogen peroxide, a reactive oxygen species released during roasting.^22^ A systematic review of prospective cohort studies on coffee consumption and the risk of CRC concluded that the evidence was inconclusive for an effect of coffee consumption on CRC risk,^23^ although some case-control studies found coffee to be protective in colon cancer but not rectal cancer.^11^

Milk and other dairy products have been hypothesized to reduce the risk of CRC through their high calcium content. Calcium is thought to protect against CRC via several biological mechanisms, including reduced cellular proliferation and promotion of cellular differentiation.^24^ Although increased milk consumption might reduce CRC risk, the evidence is not convincing.^5^

Data regarding the effects of tea, coffee, and milk on CRC risk are inconsistent. This inconsistency may be partially due to failure to distinguish among CRC subsites. Prior work suggests that there are epidemiologic and etiologic differences between colon and rectal cancers,^25^ as well as epidemiologic and etiologic differences between cancer arising in the proximal colon and in the distal colon.^26^ Few previous studies of CRC have distinguished among the different origin sites of cancers in the colorectum, even though tumors in the proximal and distal colon develop along different pathways^26^^,^^27^ and the effects of some risk factors vary by subsite within the colorectum.^26^^,^^28^ We investigated the associations of tea, coffee, and milk consumption with CRC risk and attempted to determine if these exposures were differentially associated with the risks of proximal colon, distal colon, and rectal cancer.

## METHODS

The Western Australian Bowel Health Study (WABOHS) was a population-based case-control study of CRC.^29^^,^^30^ The cases were histologically confirmed incident cases of CRC, aged 40 to 79 years, that were reported to the Western Australian Cancer Registry between June 1, 2005 and August 31, 2007. Controls had no previous diagnosis of CRC and were randomly selected from the electoral roll by age (5-year age group) and sex strata, based upon the distribution of CRC cases in Western Australia in 2002. In total, 1544 eligible cases were invited to participate and 918 agreed (59.5%). Of the 2198 eligible controls invited, 1021 (46.5%) agreed to participate.

Information on tumor site was obtained for each case from the pathology report stored at the Western Australian Cancer Registry. Tumors arising in the cecum, ascending colon, hepatic flexure, and transverse colon were classified as proximal colon cancers; tumors arising in the splenic flexure, descending colon, and sigmoid colon were classified as distal colon cancers; and tumors arising in the rectosigmoid junction and rectum were classified as rectal cancers. Ethics approval for the WABOHS was obtained from the Human Research Ethics Committee at The University of Western Australia and the Western Australian Department of Health Confidentiality of the Health Information Committee.

Data on tea and coffee consumption 10 years previously were collected by self-administered questionnaire. The questions were adapted from the Arizona Tea Questionnaire,^31^ which was shown to have high test-retest reliability (intraclass correlation coefficients, approximately 0.7) and high relative validity (intraclass correlation coefficient = 0.77) when compared with 4-day food records.^31^ Data on frequency of consumption were collected for hot black tea, tea with milk, hot green tea, hot herbal tea, hot caffeinated coffee, hot decaffeinated coffee, and iced coffee. Tea with milk and hot caffeinated coffee were both classified into 4 categories: none, less than 1 cup/day, 1 cup/day, and 2 or more cups/day. Black tea was classified into 3 categories (none, less than 1 cup/day, and 1 or more cups/day), while green tea, herbal tea, decaffeinated coffee, and iced coffee were all categorized as none, less than 1 cup/month, less than 1 cup/week, and 1 or more cups/week.

Data on food intake (including milk) 10 years previously were collected using a validated 74-item food frequency questionnaire developed by the Cancer Council of Victoria,^32^ which was found to be as reliable as other questionnaires designed to measure recent dietary intake.^33^ Frequency of consumption data were collected for total milk intake (including flavored milk and milk added to tea and coffee). Total milk intake was categorized as less than 1 cup/day, 1 cup/day, and 2 or more cups/day. Data from the food frequency questionnaire were used by the Cancer Council of Victoria to calculate daily intake of foods, specific food nutrients, and total energy, based on Australian Food Composition Tables.^34^

Participants in the WABOHS were also asked about demographic information, smoking, physical activity, medication use, and vitamin supplement use.

### Statistical analysis

We excluded from the analysis all participants with missing dietary data (*n* = 3), inconsistent information on their food frequency questionnaire regarding fruit or vegetable intake (*n* = 18), no classification of cancer site (*n* = 6), and extreme energy intakes (*n* = 76). Extreme energy intake was defined as less than 500 kcal/day or greater than 3500 kcal/day for women, and less than 800 kcal/day or greater than 4000 kcal/day for men.^35^ A further 34 participants were dropped because of missing values for the potential confounders physical activity (*n* = 10), diabetes (*n* = 4), socioeconomic status (*n* = 14), and country of birth (*n* = 6). This left 948 controls (556 men, 392 women) and 854 cases (281 with proximal colon cancer [152 men, 129 women], 260 with distal colon cancer [160 men, 100 women], and 313 with rectal cancer [213 men, 100 women]) available for this analysis.

Logistic regression was used to assess the associations of tea, coffee and milk consumption with CRC risk, and multinomial logistic regression was used to determine if the associations differed for cancers of the proximal colon, distal colon, and rectum. Because the controls were frequency-matched to the cases by age and sex, these variables were included in all models. Energy intake, multivitamin use, alcohol consumption, physical activity, body mass index at age 40 years, smoking, diabetes, socioeconomic status, and country of birth were considered potential confounders, based on previous studies.^36^ We also assessed the influence of fruit consumption, vegetable consumption, meat consumption, multivitamin use, and dietary intake of folate, fiber, sugars, and saturated fat on the results; however, these variables had virtually no effect on the risk estimates and were thus not included in the final analyses. All adjustment variables were treated as categorical variables except for energy intake, which was continuous. All tea, coffee, and milk variables were mutually adjusted. Tests for trend were performed by treating dietary intakes of all food groups as continuous variables.

The proportion of missing data was 8.7% for BMI at age 40 years (Table 1) and generally less than 1% for the other variables. To account for this in the fully adjusted models, missing BMI data were imputed using the multiple imputation command, *ice*.^37^ All the exposure and outcome variables included in the final model, as well as height, maximum weight, and weight in the previous year, at age 20 years, and at age 60 years, were included in the imputation procedure.

**Table 1.** Distribution of sociodemographic, lifestyle, and dietary characteristics among cases and controls in the Western Australian Bowel Health Study, 2005–2007

	Cases (*n* = 854)	Controls (*n* = 948)
Sex		
Male (%)	61.6	58.6
Female (%)	38.4	41.4
Age (years)		
Mean ± SD	65.0 ± 8.9	64.6 ± 9.4
Age group		
40–49 years (%)	4.8	7.0
50–59 years (%)	24.1	21.8
60–69 years (%)	36.4	35.2
70–79 years (%)	34.7	36.0
Energy intake (kJ)		
Mean ± SD	8636 ± 2772	8338 ± 2764
Range	2278–16 664	2298–16 714
Multivitamin use		
Yes (%)	34.4	39.8
No (%)	65.6	60.2
Alcohol consumption (grams/day)		
<10 (%)	48.1	51.1
10–19.9 (%)	13.3	15.6
20–29.9 (%)	12.4	11.2
≥30 (%)	26.1	22.2
Physical activity at age 19–34 years		
<150 min/week (%)	50.4	50.4
≥150 min/week (%)	49.6	49.6
Smoking		
Never (%)	39.8	45.8
Former (%)	51.1	44.6
Current (%)	9.1	9.6
Diabetes		
Neither (%)	79.4	85.8
High blood sugar levels only (%)	5.6	5.2
Non-insulin dependent (%)	12.6	7.8
Insulin dependent (%)	2.3	1.3
Socioeconomic status		
Quintile 1 – Lowest (%)	24.9	19.6
Quintile 2 (%)	16.4	20.6
Quintile 3 (%)	19.0	19.5
Quintile 4 (%)	21.1	20.0
Quintile 5 – Highest (%)	18.6	20.3
Country of birth		
Australia/New Zealand (%)	62.2	66.9
Europe (%)	31.6	28.6
Other (%)	6.2	4.5
Body mass index at age 40 years		
<25 (%)	48.4	53.4
25.0–29.9 (%)	30.9	29.3
≥30 (%)	11.8	8.6
Missing (%)	8.9	8.6

There was no significant interaction between sex and any of the exposure variables (*P* > 0.1), so men and women were combined in all analyses. Differences in the beta estimate for each risk factor across colorectal subsites were tested using the post-estimation commands written by Long and Freese.^38^ Data were analyzed using Stata 11.1 (StataCorp, College Station, TX, USA).

## RESULTS

Cases were more likely than controls to have received a diagnosis of diabetes, resided in areas with lower socioeconomic status, consumed more than 30 grams of alcohol per day, and have been overweight or obese at age 40 years (Table 1). Mean energy intake was also higher among cases than among controls. Cases were less likely than controls to have never smoked, to have taken multivitamins, and to have been born in Australia or New Zealand.

The adjusted odds ratios (AORs) and 95% CIs for the associations of each tea, coffee, and milk type consumed 10 years previously with CRC risk are summarized in Table 2; subsite-specific results are summarized in Table 3. Consumption of black tea with or without milk was associated with a slight increase in CRC risk at all sites, although none of the risk increases were statistically significant and there was no indication of a dose-response relationship.

**Table 2.** Associations of tea, coffee, and milk consumption with colorectal cancer risk in the Western Australian Bowel Health Study, 2005–2007

Consumption variable (10 years previously)	Cases (men/women)	Controls (men/women)	AOR (95% CI)^a^
Black tea			
None	583 (358/225)	647 (381/266)	1.00
<1 cup/day	138 (83/55)	159 (98/61)	0.96 (0.73–1.28)
≥1 cup/day	133 (84/49)	142 (77/65)	1.29 (0.94–1.77)
*P trend*			*0.196*
Tea with milk			
None	258 (157/101)	317 (172/145)	1.00
<1 cup/day	159 (111/47)	155 (100/55)	1.34 (0.98–1.82)
1 cup/day	191 (107/84)	201 (111/90)	1.33 (0.98–1.79)
≥2 cups/day	247 (150/97)	275 (173/102)	1.22 (0.91–1.64)
*P trend*			*0.218*
Green tea			
None	739 (469/270)	811 (489/322)	
<1 cup/month	51 (26/25)	59 (34/25)	0.99 (0.64–1.52)
<1 cup/week	24 (13/11)	25 (14/11)	1.15 (0.62–2.13)
≥1 cup/week	40 (17/23)	53 (19/34)	0.99 (0.62–1.58)
*P trend*			*0.920*
Herbal tea			
None	720 (480/340)	769 (481/288)	1.00
<1 cup/month	46 (16/30)	54 (26/28)	1.00 (0.63–1.57)
<1 cup/week	43 (17/26)	48 (27/21)	1.01 (0.63–1.60)
≥1 cup/week	45 (12/33)	77 (22/55)	0.69 (0.45–1.05)
*P trend*			*0.149*
Hot coffee			
None	71 (42/29)	101 (52/49)	1.00
<1 cup/day	273 (165/108)	312 (178/134)	1.22 (0.85–1.75)
1 cup/day	300 (182/118)	312 (184/128)	1.36 (0.95–1.96)
≥2 cups/day	210 (136/74)	223 (142/81)	1.24 (0.84–1.84)
*P trend*			*0.295*
Iced coffee			
None	512 (325/187)	590 (371/219)	1.00
<1 cup/month	158 (91/67)	210 (101/109)	0.89 (0.69–1.15)
<1 cup/week	123 (69/54)	88 (48/40)	1.64 (1.20–2.24)
≥1 cup/week	61 (40/21)	60 (36/24)	1.19 (0.80–1.76)
*P trend*			*0.035*
Decaffeinated coffee			
None	721 (459/262)	786 (468/318)	1.00
<1 cup/month	39 (19/20)	65 (36/29)	0.68 (0.44–1.05)
<1 cup/week	28 (16/12)	25 (15/10)	1.28 (0.72–2.27)
≥1 cup/week	66 (31/35)	72 (37/35)	1.14 (0.79–1.65)
*P trend*			*0.536*
Total milk intake			
<1 cup/day	436 (282/154)	501 (302/199)	1.00
1 cup/day	335 (189/146)	364 (202/162)	1.03 (0.83–1.27)
≥2 cups/day	83 (54/29)	83 (52/31)	1.02 (0.71–1.46)
*P trend*			*0.850*

AOR = Adjusted Odds Ratio.

^a^Adjusted for age group, sex, energy intake from food, alcohol intake, smoking status, use of multivitamins, diabetes, physical activity during the age period 19–34 years, body mass index at age 40 years, socioeconomic status, and country of birth.

**Table 3.** Associations of tea, coffee, and milk consumption with risks of proximal and distal colon cancer and rectal cancer in the Western Australian Bowel Health Study, 2005–2007

Consumption Variable (10 years previously)	Proximal colon (*n* = 281)		Distal colon (*n* = 260)		Rectum (*n* = 313)	
		
	*n*	AOR (95% CI)^a^	*n*	AOR (95% CI)^a^	*n*	AOR (95% CI)^a^
Black tea						
None	191	1.00	177	1.00	215	1.00
<1 cup/day	43	0.91 (0.60–1.37)	46	1.09 (0.72–1.66)	49	0.91 (0.61–1.35)
≥1 cup/day	47	1.24 (0.79–1.93)	37	1.33 (0.82–2.15)	49	1.31 (0.85–2.04)
*P trend*		*0.486*		*0.254*		*0.364*
Tea with milk						
None	90	1.00	77	1.00	91	1.00
<1 cup/day	45	1.15 (0.73–1.80)	53	1.48 (0.94–2.33)	60	1.38 (0.90–2.12)
1 cup/day	62	1.15 (0.75–1.76)	61	1.49 (0.95–2.32)	68	1.36 (0.90–2.07)
≥2 cups/day	84	1.12 (0.74–1.70)	69	1.28 (0.82–2.01)	94	1.30 (0.86–1.95)
*P trend*		*0.611*		*0.298*		*0.250*
Green tea						
None	240	1.00	226	1.00	273	1.00
<1 cup/month	21	1.36 (0.76–2.41)	14	0.75 (0.38–1.46)	16	0.91 (0.48–1.71)
<1 cup/week	4	0.61 (0.20–1.83)	9	1.31 (0.55–3.10)	11	1.49 (0.67–3.32)
≥1 cup/week	16	0.95 (0.50–1.78)	11	0.97 (0.47–2.03)	13	1.05 (0.53–2.10)
*P trend*		*0.837*		*0.975*		*0.670*
Herbal tea						
None	230	1.00	222	1.00	268	1.00
<1 cup/month	15	0.94 (0.49–1.82)	16	1.17 (0.61–2.27)	15	0.90 (0.46–1.75)
<1 cup/week	15	1.11 (0.58–2.13)	14	0.97 (0.49–1.90)	14	0.94 (0.48–1.84)
≥1 cup/week	21	0.95 (0.54–1.66)^b^	8	0.37 (0.16–0.82)^b^	16	0.73 (0.39–1.35)
*P trend*		*0.966*		*0.044*		*0.329*
Hot coffee						
None	27	1.00	16	1.00	28	1.00
<1 cup/day	93	1.11 (0.67–1.83)	80	1.55 (0.85–2.84)	100	1.11 (0.67–1.84)
1 cup/day	93	1.11 (0.67–1.85)	95	1.91 (1.05–3.48)	112	1.28 (0.78–2.11)
≥2 cups/day	68	1.11 (0.64–1.91)	69	1.78 (0.94–3.36)	73	1.05 (0.61–1.81)
*P trend*		*0.780*		*0.085*		*0.790*
Iced coffee						
None	168	1.00	164	1.00	180	1.00
<1 cup/month	62	1.01 (0.71–1.44)	43	0.74 (0.50–1.11)	53	0.89 (0.61–1.28)
<1 cup/week	32	1.23 (0.78–1.95)	39	1.63 (1.04–2.54)	52	2.06 (1.37–3.10)
≥1 cup/week	19	1.11 (0.63–1.96)	14	0.86 (0.46–1.63)	28	1.52 (0.91–2.54)
*P trend*		*0.474*		*0.551*		*0.004*
Decaffeinated coffee						
None	234	1.00	223	1.00	264	1.00
<1 cup/month	10	0.51 (0.25–1.03)	15	0.84 (0.45–1.57)	14	0.72 (0.39–1.36)
<1 cup/week	11	1.51 (0.70–3.22)	4	0.58 (0.19–1.75)	13	1.69 (0.82–3.51)
≥1 cup/week	26	1.19 (0.73–1.94)	18	1.16 (0.66–2.03)	22	1.06 (0.63–1.80)
*P trend*		*0.515*		*0.978*		*0.604*
Total milk intake						
<1 cup/day	134	1.00	143	1.00	159	1.00
1 cup/day	122	1.20 (0.88–1.62)	96	0.91 (0.66–1.25)	117	0.99 (0.74–1.34)
≥2 cups/day	25	0.96 (0.57–1.62)	21	0.85 (0.49–1.49)	37	1.24 (0.77–1.98)
*P trend*		*0.639*		*0.474*		*0.522*

AOR = Adjusted Odds Ratio.

^a^Adjusted for age group, sex, energy intake from food, alcohol intake, smoking status, use of multivitamins, diabetes, physical activity during the age period 19–34 years, body mass index at age 40 years, socioeconomic status, and country of birth.

^b^*P*-value for difference between proximal colon and distal colon is <0.05.

Participants who consumed 1 or more cups of herbal tea a week 10 years previously had a significantly reduced risk of distal colon cancer as compared with participants who consumed no herbal tea (AOR, 0.37; 95% CI, 0.16–0.82; Table 3). Herbal tea consumption was not associated with the risks of proximal colon cancer or rectal cancer, but the effect of herbal tea differed significantly between the proximal and distal colon (*P* = 0.04). Green tea was not associated with total or subsite-specific CRC risk (Tables 2 and 3).

Hot coffee consumption was not significantly associated with total CRC risk (Table 2), although elevated risks were seen for distal colon cancer (Table 3). Participants who consumed some iced coffee, but less than 1 cup per week, had an increased risk of distal colon cancer (AOR, 1.63; 95% CI, 1.04–2.54) and rectal cancer (AOR, 2.06; 95% CI, 1.37–3.10) as compared with those who consumed none; however, consumption of 1 or more cups per week was only associated with a nonsignificant increase in rectal cancer risk (AOR, 1.52; 95% CI, 0.91–2.54). Decaffeinated coffee and total milk intake were not significantly associated with total or subsite-specific CRC risk (Tables 2 and 3).

Except where mentioned above, none of the beta estimates for any of the exposure variables significantly differed across colorectal subsites.

## DISCUSSION

The results of this study suggest that consumption of herbal tea and iced coffee may be associated with the risk of cancers arising in different parts of the colorectum. Consumption of herbal tea was associated with a significantly decreased risk of distal colon cancer, and consumption of iced coffee was associated with a significantly increased risk of rectal cancer. However, neither herbal tea consumption nor iced coffee consumption was associated with the risk of proximal colon cancer. Hot caffeinated coffee was associated with a possible increased risk of distal colon cancer. Black tea (with or without milk), green tea, decaffeinated coffee, and milk were not significantly associated with CRC risk at any subsite.

The results of this study suggest a possible inverse association between herbal tea and distal colon cancer risk. However, this finding is difficult to interpret, as herbal tea is a broad term used to describe many different hot teas, such as peppermint, chamomile, rooibos, and ginger teas. Information on the type of herbal tea consumed was not collected in this study. One possibility for this finding is that people who drink herbal tea may be more health conscious than those do not; however, adjusting for lifestyle factors such as physical activity, smoking, and alcohol intake did not affect the inverse association between herbal tea and the risk of distal colon cancer. It is also possible that the association was a chance finding. Two previous case-control studies found an inverse association between herbal tea and breast cancer risk,^15^^,^^16^ which suggests that the association between specific herbal teas and cancer risk is an area that warrants further research.

Consumption of iced coffee was significantly associated with increased risk of rectal cancer in this study population. In Australia, iced coffee is typically pre-made with sugar and milk. However, hot coffee, decaffeinated coffee, and milk were not associated with rectal cancer risk in this study, which suggests the positive association with iced coffee is a chance association. Another possibility is that the sugar in iced coffee increases rectal cancer risk, as iced coffee contains substantially more sugar than does hot coffee, and there is suggestive evidence that consumption of foods containing high amounts of sugar is associated with increased CRC risk.^5^

Hot coffee was associated with increased CRC risk in this study, although in subsite-specific analyses the increased risk was seen only for distal colon cancer. Other studies have reported elevated risk estimates for the association between coffee and CRC^39^^–^^41^; however, this finding is inconsistent with much of the previous literature. A meta-analysis of case-control studies found a modest inverse association between coffee consumption and CRC, although a high degree of heterogeneity was observed in the included studies.^42^ One possible reason for our somewhat inconsistent finding is that most case-control studies of coffee and CRC risk have been conducted in Europe, where the methods used to prepare and brew coffee may differ from those used in other parts of the world.^43^ A meta-analysis of cohort studies found no significant association between coffee consumption and CRC risk,^23^ suggesting that coffee consumption does not influence the development of CRC and that the increased risk found in this study may be a chance finding. Of the previous studies that examined the effect of coffee on subsite-specific CRC risk,^44^^–^^48^ only 1 found that coffee had a differential effect on the risks of proximal colon and distal colon cancers,^44^ and none found a significant association between coffee and rectal cancer risk.^44^^–^^47^ Given the results of previous research, it is likely that the possible subsite differences observed in this study are due to chance. Decaffeinated coffee intake was not associated with CRC risk in this study.

Black tea (with or without milk), green tea, and milk were not significantly associated with CRC risk, regardless of subsite. The lack of a significant association between black tea and CRC risk in this study is consistent with previous research,^49^ as is our finding that the effect of black tea on colon cancer does not differ by subsite (proximal vs distal colon).^44^^,^^46^^,^^48^^,^^50^ Our finding that green tea was not associated with CRC risk is consistent with cohort studies of this issue, although some case-control studies found a significant risk reduction.^49^ Although previous studies found a modest reduction in CRC risk with milk intake (10% decreased risk per 200 grams consumed per day),^5^^,^^51^ we found no association between milk intake and CRC risk in this study, possibly because the small number of participants for each CRC subsite in this study resulted in insufficient statistical power to detect an association of this magnitude.

The genetic, and morphologic differences between proximal and distal colon cancers may result in differential susceptibility to environmental risk factors. However, while there are no obvious mechanistic reasons why consumption of the presently investigated beverages should have differential effects on the risk of cancers arising in different parts of the colon, three possible mechanisms are polyphenols, N-nitroso compounds, and bile acids. A recent study found that polyphenol intake was differentially associated with the risks of proximal and distal colon cancer.^52^ Polyphenols in teas and coffee may reduce cancer risk by blocking endogenous formation of N-nitroso compounds,^53^ and research suggests that markers of N-nitroso compounds differ by colorectal subsite.^54^ Consumption of caffeinated beverages may reduce CRC risk by reducing secretion of bile acids,^43^ and research indicates that metabolism of bile acids may differ in the proximal and distal colon.^55^ Further epidemiologic research is needed to determine whether the beverages investigated in this study have different effects on the risk of cancers arising in different parts of the colorectum, and further mechanistic evidence is needed to understand why this may be the case.

This study had several limitations. The prevalence of the highest level of several of the exposures was less than 10%, and the small number of participants in some categories of the subsite-specific analyses resulted in insufficient power to detect small increases or decreases in risk associated with some of the exposures. For example, a post-hoc power calculation for herbal tea indicated that we had 83% power (with 8.1% exposure among controls and 3 controls per case) to detect the observed odds ratio of 0.37 for distal colon cancer but only 20% power to detect the odds ratio of 0.73 associated with rectal cancer. Also, the low proportion of participants who regularly consumed (ie, >1 cup per week) green tea, herbal tea, or decaffeinated coffee meant that we were unable to investigate whether higher consumption of these beverages was associated with overall or subsite-specific CRC risk in this study population. Research in populations with higher consumption of these beverages is needed in order to improve understanding of their effects on CRC risk. 

The food frequency questionnaire used in this study asked about dietary intake 10 years previously, which increases the chance of recall bias. However, we do not expect that recall would be differential between cases and controls. Asking participants to recall their dietary intake 10 years before may also have increased the likelihood of exposure misclassification, which may have biased the risk estimates towards the null. However, the food frequency questionnaire used in this study was found to be as reliable as other questionnaires designed to measure recent dietary intake.^33^ It is also possible that 10 years may not have been the appropriate latency period. Finally, while response fractions of 59.5% and 46.5% for cases and controls, respectively, increase the potential for selection bias, there is no reason to believe that tea, coffee, or milk consumption would have necessarily influenced participation in this study.

One strength of this study was the use of pathology reports for accurate determination of site of cancer origin in the large bowel. A further strength of this study was the measurement of tea and coffee, which included the type consumed, frequency of consumption, and amount of consumption. However, the exposure assessment used in this study may not accurately reflect tea flavonoid consumption or intakes of Maillard reaction products, cafestol, or kahweol in coffee, that is, the antioxidants and potential anticarcinogens that reach the colon and rectum. It has been suggested that research in this area should collect information on preparation (eg, hot, iced, brew time, brew strength/concentration), volume consumed per serving, and tea or coffee type (eg, green, oolong, black, caffeinated/decaffeinated, filtered/unfiltered), as these factors influence antioxidant bioavailability in the colon and rectum.^8^

In conclusion, this study found that consumption of black tea (with or without milk), green tea, decaffeinated coffee, and milk were not significantly associated with CRC risk. Hot coffee was associated with a possible increased risk of distal colon cancer; however, this finding is inconsistent with much of the previous literature. An association between iced coffee and increased risk of rectal cancer was internally inconsistent and may be a chance finding. Herbal tea consumption was associated with a significantly decreased risk of distal colon cancer, and this relationship may warrant further investigation.
